# Prediction model for tibial plateau fracture combined with meniscus injury

**DOI:** 10.3389/fsurg.2023.1095961

**Published:** 2023-06-16

**Authors:** Hongzhi Lv, Wenjing Li, Yan Wang, Wei Chen, Xiaoli Yan, Peizhi Yuwen, Zhiyong Hou, Juan Wang, Yingze Zhang

**Affiliations:** Department of Orthopedic Surgery, The Third Hospital of Hebei Medical University, Shijiazhuang China

**Keywords:** tibial plateau fracture, meniscus injury, risk factors, prediction model, nomogram

## Abstract

**Purpose:**

To investigate a prediction model of meniscus injury in patients with tibial plateau fracture.

**Methods:**

This retrospective study enrolled patients with tibial plateau fractures who were treated in the Third Hospital of Hebei Medical University from January 1, 2015, to June 30, 2022. Patients were divided into a development cohort and a validation cohort based on the time-lapse validation method. Patients in each cohort were divided into a group with meniscus injury and a group without meniscus injury. Statistical analysis with Student’s t-test for continuous variables and chi square test for categorical variables was performed for patients with and without meniscus injury in the development cohort. Multivariate logistic regression analysis was used to screen the risk factors of tibial plateau combined with meniscal injury, and a clinical prediction model was constructed. Model performance was measured by examining discrimination (Harrell’s C-index), calibration (calibration plots), and utility [decision analysis curves (DCA)]. The model was validated internally using bootstrapping and externally by calculating their performance in a validation cohort.

**Results:**

Five hundred patients (313 [62.6%] males, 187 [37.4%] females) with a mean age of 47.7 ± 13.8 years were eligible and were divided into development (*n* = 262) and validation (*n* = 238) cohorts. A total of 284 patients had meniscus injury, including 136 in the development cohort and 148 in the validation cohort We identified high-energy injuries as a risk factor (OR* *= 1.969, 95%CI 1.131–3.427). Compared with blood type A, patients with blood type B were more likely to experience tibial plateau fracture with meniscus injury (OR* *= 2.967, 95%CI 1.531–5.748), and office work was a protective factor (OR* *= 0.279, 95%CI 0.126–0.618). The C-index of the overall survival model was 0.687 (95% CI, 0.623–0.751). Similar C-indices were obtained for external validation [0.700(0.631–0.768)] and internal validation [0.639 (0.638–0.643)]. The model was adequately calibrated and its predictions correlated with the observed outcomes. The DCA curve showed that the model had the best clinical validity when the threshold probability was 0.40 and 0.82.

**Conclusions:**

Patients with blood type B and high-energy injuries are more likely to have meniscal injury. This may help in clinical trial design and individual clinical decision-making.

## Introduction

The meniscus is an important fibrocartilage structure in the knee joint that functions as a lubricant and cushion, minimizing shock, and stress in the joint (1, 2). Tibial plateau fracture is a complex traumatic fracture caused by the stress of external or internal rotation of the tibia, combined with the axial force of knee flexion (3–5). Fractures are often accompanied by meniscal injury, with an incidence of 39%–99% (6). At present, resection is often used to treat meniscal injury, though this reduces the contact area between the meniscus and tibial femur, increases contact pressure, and reduces joint stability. This leads to pain and limitation of knee function, and can even cause articular cartilage degeneration and osteoarthritis (7, 8). Therefore, the integrity of the meniscus structure is important for the function and quality of life in patients with tibial plateau fractures. Therefore, it is crucial for clinical prevention to predict the high-risk population for tibial plateau fractures combined with meniscal injury.

The clinical prediction model, known as the clinical prediction rule or risk score, refers to the use of multifactorial models to predict the probability of having a certain disease or certain future outcome. Nomograms (also known as nomograms) are graphical descriptions of models that estimate the probability of an event occurring for an individual patient (9, 10). The precise prevention and control of diseases is important. In terms of predicting the risk factors of tibial plateau fracture combined with meniscal injury, previous studies have only analyzed the risk factors of tibial plateau fracture or meniscus injury alone (11–13). There are still no models that adequately predict meniscal injuries in patients with tibial plateau fractures.

Therefore, we designed this study to retrospectively collect the data of patients with tibial plateau fractures admitted to the Third Hospital of Hebei Medical University from January 2015 to June 2022. We aimed to describe and analyze the demographic characteristics and preoperative fracture-related factors and develop nomograms to predict the possibility of meniscal injury in these patients. This study provides a reference for clinical diagnosis, treatment, and disease prevention.

## Participants and methods

### Inclusion criteria and exclusion criteria

The study inclusion criteria were as follows: (i) closed tibial plateau fractures and (ii) complete medical records and imaging data. The exclusion criteria were as follows: (i) pathologic fractures, (ii) old or secondary tibial plateau fractures, (iii) open tibial plateau fractures, and (iv) incomplete data and imaging material.

### Study design

The clinical data of patients with tibial plateau fractures admitted to the Third Hospital of Hebei Medical University from January 1, 2015, to June 30, 2022, was retrospectively analyzed. Fracture patients admitted between January 2019 and June 2022 were assigned as the development cohort to analyze risk factors for meniscus injury and built a nomogram, and patients admitted between January 2015 and December 2018 were assigned as the validation cohort to evaluate the transportability and generalizability of the model. This epidemiological study was approved by the Institutional Review Board of the Third Hospital of Hebei Medical University (section 2015-002-1) in compliance with the Helsinki Declaration. This was a retrospective study based on historical medical records, and no human participants were included. Written informed consent was not required for participation.

### Data collection

The medical records of enrolled patients were retrieved using a medical record inquiry system. The demographic characteristics of the patients and detailed information on the tibial plateau fractures were recorded. Specifically, collated information included (1) demographic characteristics: sex, age, marital status, occupation, ethnic origin, body mass index (BMI), medical payment method, urbanization, and blood type; (2) injury characteristics: season, holiday, injury mechanism, injury side, AO classification, Schatzker classification, ligament injury, and associated injuries; (3) preoperative complications and preoperative concomitant injuries.

Preoperative and postoperative imaging data were collected using a picture archiving and communication system. x-ray, computed tomography (CT) and magnetic resonance imaging (MRI) scans were performed on all patients with tibial plateau fracture meeting the inclusion and exclusion criteria after admission. X-ray films, CT and MRI scans, and arthroscopy of tibial plateau fractures were reviewed by two orthopaedic surgeons with more than 10 years of experience. Schatzker and AO fracture classification were used to assess the pattern of tibial plateau fractures. If there was any disagreement in the classification of tibial plateau fractures and the diagnosis of meniscus and ligament damage, a final decision was made through discussion, with consensus achieved by the two surgeons.

### Surgical procedures

All 500 patients with tibial plateau fractures underwent surgical treatment. The patients with Schatzker type I split of the lateral tibial plateau was treated with prepatellar clamp reduction and plate screw fixation. Type Ⅱ and Ⅲ split of the lateral tibial plateau combined with collapse and simple collapse fracture were treated with Kirschner wire pry reduction, arthroscopic reduction and plate screw fixation, or balloon distraction and bone cement fixation. For type Ⅳ medial tibial plateau fracture, the anterior patellar clamp was used for reduction, Kirschner wire pry for reduction, and medial plate screw fixation. For type Ⅴ and Ⅵ bilateral tibial plateau fractures, the collapsed bone was reduced by incised Kirschner wire pry, the width of the tibial plateau was restored by prepatellar forceps, and bilateral plate screws were used for fixation. In addition to fracture reduction and fixation, we performed arthroscopy during the operation. On the one hand, we observed whether the reduction quality was satisfactory. At the same time, we explored whether the anterior and posterior cruciate ligaments and meniscus were damaged or torn. and intraoperative arthroscopy was performed to observe whether the reduction quality was satisfactory and whether there were anterior and posterior cruciate ligaments and meniscus injuries.

### Statistical analysis

Statistical analyses were performed using R4.1.0 (R Foundation for Statistical Computing, Austria). Statistical significance was set at *P* < 0.05. Descriptive statistics were reported as frequencies and proportions, cause the collected factors were all categorical variable. Differences in the constituent ratios of baseline comparison of tibial plateau fractures between the development and validation cohorts were tested using the chi squared (*χ*^2^*)* test or Fisher’s exact probability method. Model building used univariate analysis to analyze the 53 variables. Variables with *P *< 0.05 were selected and included in multivariate logistic regression for further analysis to obtain the independent influencing factors related to tibial plateau fracture combined with meniscal injury and a nomogram plot were established. Logistic regression analyses were used to estimate the odds ratios (OR) and their 95% confidence intervals (CIs) or *P*-values. Model performance was evaluated by examining discrimination (C-index), calibration (calibration plots and H-L goodness of fit test), and utility [decision analysis curves (DCA)].

The model validation was performed in two steps. First, we performed an internal validation using a bootstrap resampling process to provide an unbiased estimate of model performance, using the C-index (validated package in R) and calibration plot (calibrate package in R). The original development cohort was resampled to obtain a dataset with the same size. Second, to assess external validity, the prediction accuracy of the model was determined in the validation cohort by computing the C-indices, calibration plots, and H-L goodness of fit test. The clinical effectiveness was evaluated in the validation cohort using the DCA curve, and the net clinical benefit of the model was obtained.

## Results

### Characteristics of fracture cases

**Figure 1:**
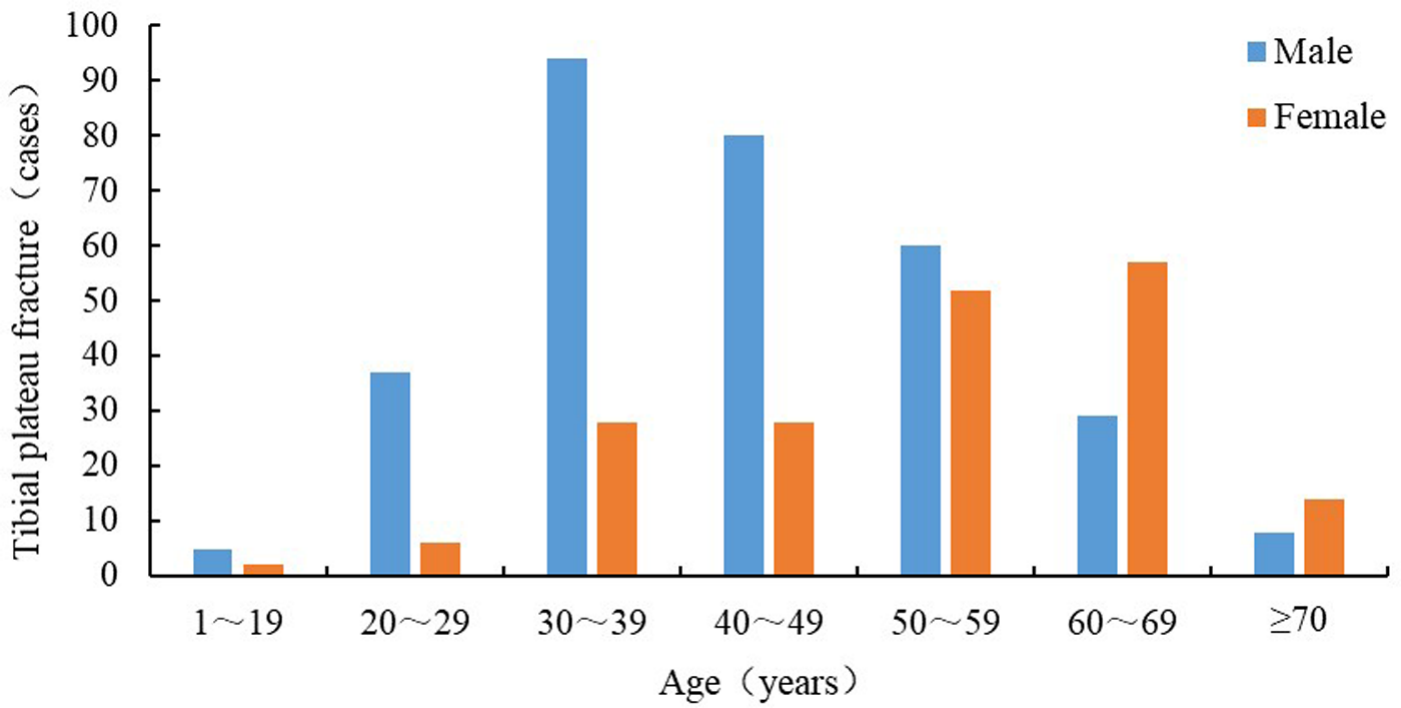
Gender and age distribution of 500 patients with tibial plateau fractures.

A total of 500 patients diagnosed with tibial plateau fracture, 313 males (62.6%) and 187 females (37.4%) with an average age of 47.7 ± 13.8 years (range, 14–89 years) met the eligibility criteria (Figure 1). Before the age of 60 years, male patients accounted for the majority of tibial plateau fractures, with men aged 30–39 years old accounting for the largest proportion (86.0%). After 60 years of age, most tibial plateau fracture patients were female, and 66.3% of them were aged 60–69 years old (*χ*^2^*^ ^*= 68.285, *P *< 0.01). There were 284 (56.8%) cases of tibial plateau fracture complicated by meniscal injury and 216 (43.2%) cases without meniscus injury. There were 84 cases (16.8%) with ligament injury, including 61 cases (72.6%) with anterior forks ligament injury, 21 cases (25.0%) with posterior forks ligament injury, and 2 cases (2.4%) with anterior forks ligament and posterior forks ligament injuries. According to the AO classification, 306 (61.8%) cases were type B fractures and 189 (38.2%) were type C fractures. According to the Schatzker classification, type II fractures were the most common (214 cases, 42.8%), followed by types VI (112, 22.4%), V (73, 14.6%), IV (56, 11.2%), III (40, 8.0%), and I (5, 1.0%). Among the 500 patients with tibial plateau fractures, the majority were overweight (266 cases, 53.2%), followed by obese (125 cases, 25.0%), normal weight (105 cases, 21.0%), and underweight (4 cases, 0.8%). The medical insurance group comprised the majority of patients (491 cases, 98.2%), and most patients were married (462 cases, 93.4%). Moreover, most of the patients were diagnosed in spring (150 cases, 30.0%), followed by winter (124 cases, 24.8%), autumn (120 cases, 24.0%), and summer (106 cases, 21.2%). Most patients were of Han nationality (488 cases, 97.6%), and from rural areas (306 cases, 61.2%). Blood type B was predominant (162 cases, 32.4%), followed by blood type A (142 cases, 28.4%), type O (139 cases, 27.8%), and type AB (57 cases, 11.4%). Hypertension was the most common preoperative complication (104 patients, 20.8%). The comparison results of the baseline data between the development and validation cohorts are shown in Table 1.

**Table 1:** Baseline comparison of tibial plateau fracture between development and validation cohort [*n* (%)].

	All	Development cohort	Validation cohort	*χ*^2^ value	*P* value
Gender				19.747	<0.001
Male	313 (62.60)	140 (53.44)	173 (72.69)		
Female	187 (37.40)	122 (46.56)	65 (27.31)		
Age(Years)				6.368	0.383
1∼19	7 (1.40)	2 (0.80)	5 (2.10)		
20∼29	43 (8.60)	18 (6.90)	25 (10.50)		
30∼39	122 (24.40)	61 (23.30)	61 (25.60)		
40∼49	108 (21.60)	55 (21.00)	53 (22.30)		
50∼59	112 (22.40)	66 (25.20)	46 (19.30)		
60∼69	86 (17.20)	47 (17.90)	39 (16.40)		
≥70	22 (4.40)	13 (5.00)	9 (3.80)		
Marital status				5.467	0.019
Unmarried	21 (4.20)	9 (3.44)	12 (5.04)		
Married	462 (93.40)	242 (92.36)	225 (94.54)		
Widowed	7 (1.40)	7 (2.67)	0 (0.00)		
Divorce	5 (1.00)	4 (1.53)	1 (0.42)		
Occupation				11.262	0.014
Farmer	178 (35.60)	81 (30.92)	97 (40.76)		
Office worker	83 (16.60)	45 (17.18)	38 (15.97)		
Manual worker	72 (14.40)	39 (14.89)	33 (13.87)		
Retired	48 (9.60)	33 (12.60)	15 (6.30)		
Unemployed	57 (11.40)	37 (14.12)	20 (8.40)		
Others	62 (12.40)	27 (10.31)	35 (14.71)		
Ethnic origin				7.600	0.006
Han	488 (97.60)	251 (95.80)	237 (99.58)		
Other	12 (2.40)	11 (4.20)	1 (0.42)		
BMI (kg/m^2^)					0.168
<18.5	4 (0.80)	3 (1.15)	1 (0.42)		
18.5∼23.9	105 (21.00)	62 (23.66)	43 (18.07)		
24.0∼27.9	266 (53.20)	128 (48.85)	138 (57.98)		
≥28.0	125 (25.00)	69 (26.34)	56 (23.53)		
Payment method				0.617	0.413
Insurance	491 (98.20)	259 (98.85)	232 (97.48)		
Self-funded	9 (1.80)	3 (1.15)	6 (2.52)		
Urbanization				0.061	0.805
Urban area	194 (38.80)	103 (39.31)	91 (38.24)		
Rural area	306 (61.20)	159 (60.69)	147 (61.76)		
Blood type				13.193	0.004
A	142 (28.40)	86 (32.82)	56 (23.53)		
B	162 (32.40)	80 (30.53)	82 (34.45)		
O	139 (27.80)	77 (29.39)	62 (26.05)		
AB	57 (11.40)	19 (7.25)	38 (15.97)		
Season				11.365	0.004
Spring	150 (30.00)	86 (32.82)	64 (26.89)		
Summer	106 (21.20)	39 (14.89)	67 (28.15)		
Autumn	120 (24.00)	69 (26.34)	51 (21.43)		
Winter	124 (24.80)	68 (25.95)	56 (23.53)		
Holiday				1.226	0.268
Yes	49 (9.80)	22 (8.40)	27 (11.34)		
No	451 (90.20)	240 (91.60)	211 (88.66)		
Injury Mechanism				3.513	0.061
High energy	303 (60.60)	169 (64.50)	134 (56.30)		
Low energy	197 (39.40)	93 (35.50)	104 (43.70)		
Side				0.581	0.446
Left	296 (59.32)	159 (60.92)	137 (57.56)		
Right	203 (40.68)	102 (39.08)	101 (42.44)		
AO classification				0.102	0.749
B	306 (61.82)	159 (61.15)	147 (62.55)		
C	189 (38.18)	101 (38.85)	88 (37.45)		
Schatzker classification				15.687	0.008
I	5 (1.00)	3 (1.10)	2 (0.80)		
II	214 (42.80)	119 (45.40)	95 (39.90)		
III	40 (8.00)	14 (5.30)	26 (10.90)		
IV	56 (11.20)	28 (10.70)	28 (11.80)		
V	73 (14.60)	49 (18.70)	24 (10.10)		
VI	112 (22.40)	49 (18.70)	63 (26.50)		
Ligament injury				0.522	0.470
Yes	84 (16.8)	41 (15.6)	43 (18.1)		
No	416 (83.2)	221 (84.4)	195 (81.9)		
Associated injuries				1.522	0.217
Yes	102 (20.40)	59 (22.52)	43 (18.07)		
No	398 (79.60)	203 (77.48)	195 (81.93)		
Complications				8.162	0.004
Yes	252 (50.40)	148 (56.49)	104 (43.70)		
No	248 (49.60)	114 (43.51)	134 (56.30)		
Hypertension				6.442	0.011
Yes	104 (20.80)	66 (25.19)	38 (15.97)		
No	396 (79.20)	196 (74.81)	200 (84.03)		
Diabetes				2.323	0.128
Yes	39 (7.80)	25 (9.54)	14 (5.88)		
No	461 (92.20)	237 (90.46)	224 (94.12)		
Coronary disease				0.032	0.859
Yes	24 (4.80)	13 (5.00)	11 (4.60)		
No	476 (95.20)	249 (95.00)	227 (95.40)		
Deep vein thrombosis				0.012	0.913
Yes	104 (20.80)	54 (20.60)	50 (21.00)		
No	396 (79.20)	208 (79.40)	188 (79.00)		
Osteoporosis				3.218	0.073
Yes	13 (2.60)	10 (3.80)	3 (1.30)		
No	487 (97.40)	252 (96.20)	235 (98.70)		
Ostarthritis					1.000
Yes	1 (0.20)	1 (0.38)	0 (0.00)		
No	499 (99.80)	261 (99.62)	238 (100.00)		
Urinary system				0.056	0.812
Yes	20 (4.00)	11 (4.20)	9 (3.78)		
No	480 (96.00)	251 (95.80)	229 (96.22)		
Hepatitis B				0.279	0.597
Yes	9 (1.80)	6 (2.29)	3 (1.26)		
No	491 (98.20)	256 (97.71)	235 (98.74)		
Cerebral infarction				9.967	0.002
Yes	18 (3.60)	16 (6.11)	2 (0.84)		
No	482 (96.40)	246 (93.89)	236 (99.16)		
Anemia				49.516	<0.001
Yes	73 (14.60)	66 (25.19)	7 (2.94)		
No	427 (85.40)	196 (74.81)	231 (97.06)		
Hypoproteinemia				30.742	<0.001
Yes	46 (9.20)	42 (16.03)	4 (1.68)		
No	454 (90.80)	220 (83.97)	234 (98.32)		
Hyponatremia				27.494	<0.001
Yes	36 (7.20)	34 (12.98)	2 (0.84)		
No	464 (92.80)	228 (87.02)	236 (99.16)		
Hypokalemia				17.512	<0.001
Yes	26 (5.20)	24 (9.16)	2 (0.84)		
No	474 (94.80)	238 (90.84)	236 (99.16)		
Hepatobiliary system				23.436	<0.001
Yes	32 (6.40)	30 (11.45)	2 (0.84)		
No	468 (93.60)	232 (88.55)	236 (99.16)		
Head injury				0.649	0.420
Yes	10 (2.00)	7 (2.67)	3 (1.26)		
No	490 (98.00)	255 (97.33)	235 (98.74)		
Facial injury				0.297	0.597
Yes	9 (1.80)	6 (2.29)	3 (1.26)		
No	491 (98.20)	256 (97.71)	235 (98.74)		
Orbital fracture				0.007	0.933
Yes	3 (0.60)	1 (0.40)	2 (0.80)		
No	497 (99.40)	261 (99.60)	236 (99.20)		
Nasal bone fracture					1.000
Yes	1 (0.20)	1 (0.38)	0 (0.00)		
No	499 (99.80)	261 (99.62)	238 (100.00)		
Pleural effusion				1.068	0.301
Yes	17 (3.40)	11 (4.20)	6 (2.52)		
No	483 (96.60)	251 (95.80)	232 (97.48)		
Lung injury				0.005	0.941
Yes	15 (3.00)	8 (3.05)	7 (2.94)		
No	485 (97.00)	254 (96.95)	231 (97.06)		
Rib fracture				3.964	0.046
Yes	30 (6.00)	21 (8.02)	9 (3.78)		
No	470 (94.00)	241 (91.98)	229 (96.22)		
Clavical fracture					1.000
Yes	4 (0.80)	2 (0.76)	2 (0.84)		
No	496 (99.20)	260 (99.24)	236 (99.16)		
Lumbar vertebrae fracture				3.903	0.048
Yes	11 (2.20)	9 (3.44)	2 (0.84)		
No	489 (97.80)	253 (96.56)	236 (99.16)		
Scapular fracture				0.048	0.826
Yes	8 (1.60)	5 (1.91)	3 (1.26)		
No	492 (98.40)	257 (98.09)	235 (98.74)		
Humeral fractures				0.627	0.428
Yes	5 (1.00)	4 (1.53)	1 (0.42)		
No	495 (99.00)	258 (98.47)	237 (99.58)		
Ulnar fracture				1.016	0.313
Yes	5 (1.00)	1 (0.38)	4 (1.68)		
No	495 (99.00)	261 (99.62)	234 (98.32)		
Radius fractures				2.847	0.092
Yes	11 (2.20)	3 (1.15)	8 (3.36)		
No	489 (97.80)	259 (98.85)	230 (96.64)		
Metacarpal fracture					1.000
Yes	3 (0.60)	2 (0.76)	1 (0.42)		
No	497 (99.40)	260 (99.24)	237 (99.58)		
Acetabular fracture				0.165	0.685
Yes	4 (0.80)	3 (1.15)	1 (0.42)		
No	496 (99.20)	259 (98.85)	237 (99.58)		
Pelvic fracture				0.048	0.826
Yes	8 (1.60)	5 (1.91)	3 (1.26)		
No	492 (98.40)	257 (98.09)	235 (98.74)		
Femoral fracture				0.048	0.826
Yes	20 (4.00)	10 (3.82)	10 (4.20)		
No	480 (96.00)	252 (96.18)	228(95.80)		
Patellar fracture				0.363	0.012
Yes	20(4.00)	16(6.10)	4(1.70)		
No	480(96.00)	246(93.90)	234(98.30)		
Calcaneal fracture				0.201	0.654
Yes	17(3.40)	8(3.05)	9(3.78)		
No	483(96.60)	254(96.95)	229(96.22)		
Meniscus injury				5.367	0.021
Yes	284(56.80)	136(51.91)	148(62.18)		
No	216(43.20)	126(48.09)	90(37.82)		

### Model building

Univariate analysis of the model building population showed that compared with the tibial plateau fracture patients without meniscus injury, the proportion of patients with meniscus injury who were farmers and workers, high-energy injury, and blood type B and blood type AB were higher (*P *< 0.05, Table 2). These factors were included in the multivariate logistic regression analysis.

**Table 2:** Results of univariate analysis of tibial plateau fracture in development cohort [case (%)].

	All	With meniscus injury	Without meniscus injury	*χ*^2^ value	*P* value
Sex				0.681	0.409
Male	140 (53.4)	76 (55.9)	64 (50.8)		
Female	122 (46.6)	60 (44.1)	62 (49.2)		
Age (years)					0.938
1∼19	2 (0.8)	1 (0.7)	1 (0.8)		
20∼29	18 (6.9)	12 (8.8)	6 (4.8)		
30∼39	61 (23.3)	30 (22.1)	31 (24.6)		
40∼49	55 (21.0)	28 (20.6)	27 (21.4)		
50∼59	66 (25.2)	34 (25.0)	32 (25.4)		
60∼69	47 (17.9)	24 (17.6)	23 (18.3)		
≥70	13 (5.0)	7 (5.1)	6 (4.8)		
Marital status				1.281	0.734
Unmarried	9 (3.4)	4 (2.9)	5 (4.0)		
Married	242 (92.4)	125 (91.9)	117 (92.9)		
Widowed	7 (2.7)	5 (3.7)	2 (1.6)		
Divorce	4 (1.5)	2 (1.5)	2 (1.6)		
Occupation				11.727	0.039*
Farmer	81 (30.9)	51 (37.5)	30 (23.8)		
Office worker	45 (17.2)	15 (11.0)	30 (23.8)		
Manual worker	39 (14.9)	23 (16.9)	16 (12.7)		
Retired	33 (12.6)	17 (12.5)	16 (12.7)		
Unemployed	37 (14.1)	18 (13.2)	19 (15.1)		
Others	27 (10.3)	12 (8.8)	15 (11.9)		
Ethnic origin				0.032	0.858
Han	251 (95.8)	130 (95.6)	121 (96.0)		
Others	11 (4.2)	6 (4.4)	5 (4.0)		
BMI (kg/m^2^)					0.501
≤18.5	3 (1.2)	2 (1.5)	1 (0.8)		
18.5∼23.9	62 (23.7)	27 (19.9)	35 (27.8)		
24.0∼27.9	128 (48.9)	69 (50.7)	59 (46.8)		
≥28.0	69 (26.3)	38 (27.9)	31 (24.6)		
Payment method				0	1.000
Insurance	259 (98.9)	134 (98.5)	125 (99.2)		
Self-funded	3 (1.2)	2 (1.5)	1 (0.8)		
Urbanization				0.138	0.711
Urban area	103 (39.3)	52 (38.2)	51 (40.5)		
Rural area	159 (60.7)	84 (61.8)	75 (59.5)		
Blood type				10.532	0.015*
A	86 (32.8)	36 (26.5)	50 (39.7)		
B	80 (30.5)	53 (39.0)	27 (21.4)		
O	77 (29.4)	37 (27.2)	40 (31.8)		
AB	19 (7.3)	10 (7.4)	9 (7.1)		
Season				0.949	0.813
Spring	86 (32.8)	43 (31.6)	43 (34.1)		
Summer	39 (14.9)	23 (16.9)	16 (12.7)		
Autumn	69 (26.3)	35 (25.7)	34 (27.0)		
Winter	68 (26.0)	35 (25.7)	33 (26.2)		
Holiday				0.496	0.481
Yes	22 (8.4)	13 (9.6)	9 (7.1)		
No	240 (91.6)	123 (90.4)	117 (92.9)		
Injury Mechanism				4.572	0.032*
High energy	169 (64.5)	96 (70.6)	73 (57.9)		
Low energy	93 (35.5)	40 (29.4)	53 (42.1)		
Side				0.677	0.411
Left	159 (60.9)	79 (58.5)	80 (63.5)		
Right	102 (39.1)	56 (41.5)	46 (36.5)		
AO classification				0.013	0.910
B	159 (61.2)	83 (61.5)	76 (60.8)		
C	101 (38.9)	52 (38.5)	49 (39.2)		
Schatzker classification				8.503	0.131
I	3 (1.2)	2 (1.5)	1 (0.8)		
II	119 (45.4)	60 (44.1)	59 (46.8)		
III	14 (5.3)	11 (8.1)	3 (2.4)		
IV	28 (10.7)	10 (7.4)	18 (14.3)		
V	49 (18.7)	29 (21.3)	20 (15.9)		
VI	49 (18.7)	24 (17.7)	25 (19.8)		
Ligament injury				1.248	0.264
Yes	41 (15.6)	18 (13.2)	23 (18.3)		
No	211 (84.4)	118 (86.8)	103 (81.7)		
Associated injuries				0.034	0.583
Yes	203 (77.5)	106 (77.9)	97 (77.0)		
No	59 (22.5)	30 (22.1)	29 (23.0)		
Complications				1.448	0.299
Yes	148 (56.5)	72 (52.9)	76 (60.3)		
No	114 (43.5)	64 (47.1)	50 (39.7)		
Hypertension				1.472	0.225
Yes	66 (25.2)	30 (22.1)	36 (28.6)		
No	196 (74.8)	106 (77.9)	90 (71.4)		
Diabetes				0.185	0.667
Yes	25 (9.54)	14 (10.29)	11 (8.7)		
No	237 (90.46)	122 (89.71)	115 (91.3)		
Coronary disease				0.021	0.886
Yes	13 (5.0)	7 (5.2)	6 (4.8)		
No	249 (95.0)	129 (94.9)	120 (95.2)		
Deep vein thrombosis				0.858	0.354
Yes	54 (20.6)	25 (18.4)	29 (23.0)		
No	208 (79.4)	111 (81.6)	97 (77.0)		
Osteoporosis				0.04	0.842
Yes	10 (3.8)	6 (4.4)	4 (3.2)		
No	252 (96.2)	130 (95.6)	122 (96.8)		
Ostarthritis					0.481
Yes	1 (0.4)	0 (0.0)	1 (0.8)		
No	261 (99.6)	136 (100.0)	125 (99.2)		
Urinary system				0.633	0.426
Yes	11 (4.2)	7 (5.2)	4 (3.2)		
No	251 (95.8)	129 (94.9)	122 (96.8)		
Hepatitis B				0.258	0.611
Yes	6 (2.3)	2 (1.5)	4 (3.2)		
No	256 (97.7)	134 (98.5)	122 (96.8)		
Cerebral infarction				0.025	0.875
Yes	16 (6.1)	8 (5.9)	8 (6.4)		
No	246 (93.9)	128 (94.1)	118 (93.7)		
Anemia				0.129	0.720
Yes	66 (25.2)	33 (24.3)	33 (26.2)		
No	196 (74.8)	103 (75.7)	93 (73.8)		
Hypoproteinemia				0.891	0.345
Yes	42 (16.0)	19 (14.0)	23 (18.3)		
No	220 (84.0)	117 (86.0)	103 (81.8)		
Hyponatremia				0.95	0.330
Yes	34 (13.0)	15 (11.0)	19 (15.1)		
No	228 (87.0)	121 (89.0)	107 (84.9)		
Hypokalemia				1.11	0.292
Yes	24 (9.2)	10 (7.4)	14 (11.1)		
No	238 (90.8)	126 (92.7)	112 (88.9)		
Hepatobiliary system				1.925	0.165
Yes	30 (11.5)	12 (8.8)	18 (14.3)		
No	232 (88.6)	124 (91.2)	108 (85.7)		
Head injury				2.048	0.152
Yes	7 (2.7)	6 (4.4)	1 (0.8)		
No	255 (97.3)	130 (95.6)	125 (99.2)		
Facial injury				0	1.000
Yes	6 (2.3)	3 (2.2)	3 (2.4)		
No	256 (97.7)	133 (97.8)	123 (97.6)		
Orbital fracture					0.481
Yes	1 (0.4)	0 (0.0)	1 (0.8)		
No	261 (99.6)	136 (100.0)	125 (99.2)		
Nasal bone fracture					1.000
Yes	1 (0.4)	1 (0.7)	0 (0.0)		
No	261 (99.6)	135 (99.3)	126 (100.0)		
Pleural effusion				1.111	0.292
Yes	11 (4.2)	4 (2.9)	7 (5.6)		
No	251 (95.8)	132 (97.1)	119 (94.4)		
Lung injury				0.938	0.333
Yes	8 (3.1)	6 (4.4)	2 (1.6)		
No	254 (97.0)	130 (95.6)	124 (98.4)		
Rib fracture				0.168	0.682
Yes	21 (8.0)	10 (7.4)	11 (8.7)		
No	241 (92.0)	126 (92.7)	115 (91.3)		
Clavical fraccture					0.499
Yes	2 (0.8)	2 (1.5)	0 (0.0)		
No	260 (99.2)	134 (98.5)	126 (100.0)		
Lumbar vertebrae fracture				3.687	0.055
Yes	9 (3.4)	8 (5.9)	1 (0.8)		
No	253 (96.6)	128 (94.1)	125 (99.2)		
Scapular fracture				0.007	0.931
Yes	5 (1.9)	2 (1.5)	3 (2.4)		
No	257 (98.1)	134 (98.5)	123 (97.6)		
Humeral fractures				0.338	0.561
Yes	4 (1.5)	1 (0.7)	3 (2.4)		
No	258 (98.5)	135 (99.3)	123 (97.6)		
Ulnar fracture					1.000
Yes	1 (0.4)	1 (0.7)	0 (0)		
No	261 (99.6)	135 (99.3)	126 (100.0)		
Radius fractures				1.201	0.273
Yes	3 (1.2)	3 (2.2)	0 (0.0)		
No	259 (98.9)	133 (97.8)	126 (100.0)		
Metacarpal fracture					1.000
Yes	2 (0.8)	1 (0.7)	1 (0.8)		
No	260 (99.2)	135 (99.3)	125 (99.2)		
Acetabular fracture				1.201	0.273
Yes	3 (1.2)	3 (2.2)	0 (0.0)		
No	259 (98.9)	133 (97.8)	126 (100.0)		
Pelvic fracture				0	1.000
Yes	5 (1.9)	3 (2.2)	2 (1.6)		
No	257(98.1)	133(97.8)	124(98.4)		
Femoral fracture				0.199	0.656
Yes	10(3.8)	4(2.9)	6(4.8)		
No	252(96.2)	132(97.1)	120(95.2)		
Patellar fracture				0.454	0.500
Yes	16(6.1)	7(5.2)	9(7.1)		
No	246(93.9)	129(94.9)	117(92.9)		
Calcaneal fracture				0	1.000
Yes	8(3.1)	4(2.9)	4(3.2)		
No	254(97.0)	132(97.1)	122(96.8)		

Multivariate analysis showed that high-energy injury (OR* *=* *1.969, 95%CI:1.131–3.427) was a risk factor for tibial plateau fracture combined with meniscal injury. Compared with blood type A, blood type B was a risk factor (OR* *= 2.967, 95%CI:1.531–5.748). Compared to farmers, office staff occupation was a protective factor (*OR *= 0.279, 95%CI:0.126–0.618) (Table 3).

**Table 3:** Results of multivariate analysis of tibial plateau fracture in development cohort.

	*B*	*S.E.*	Wald *χ*^2^value	*P* value	*OR*	95%*CI*
Injury Mechanism	0.677	0.283	5.742	0.017	1.969	1.131, 3.427
Occupation						
Farmer			10.745	0.057		
Office worker	−1.276	0.405	9.923	0.002	0.279	0.126, 0.618
Manual worker	−0.298	0.414	0.516	0.472	0.743	0.330, 1.672
Retired	−0.479	0.435	1.210	0.271	0.620	0.264, 1.454
Unemployed	−0.631	0.417	2.286	0.131	0.532	0.235, 1.205
Others	−0.693	0.467	2.200	0.138	0.500	0.200, 1.249
Blood type						
A			11.752	0.008		
B	1.087	0.337	10.383	0.001	2.967	1.531, 5.748
O	0.135	0.332	0.166	0.683	1.145	0.597, 2.195
AB	0.444	0.528	0.710	0.400	1.560	0.555, 4.387

### Model validation

**Figure 2:**
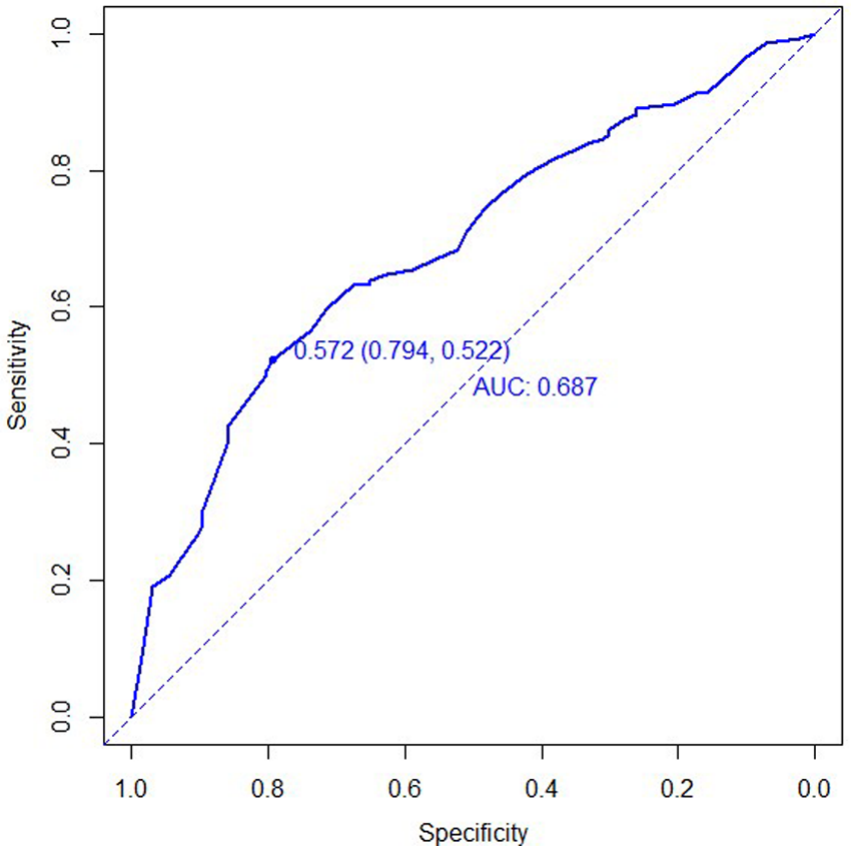
ROC curve for development cohort of tibial plateau fracture combined with meniscus injury.

**Figure 3:**
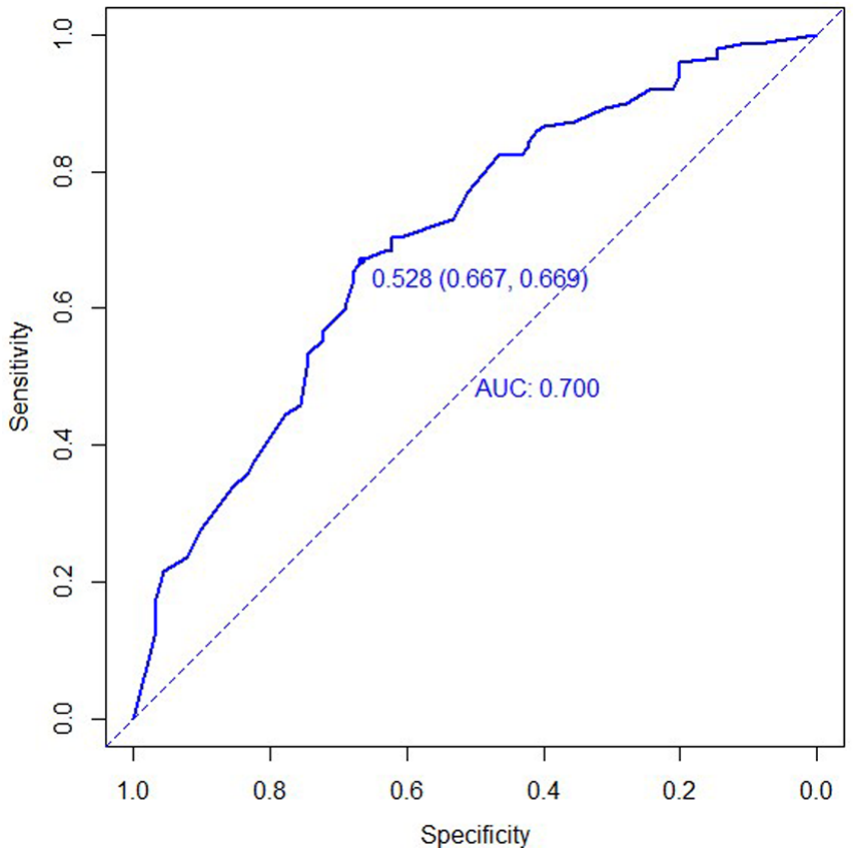
ROC curve for validation cohort of tibial plateau fracture combined with meniscus injury.

According to the ROC curve, the C-index of the model was 0.687 (95%CI:0.623–0.751), and the best cut-off value was 0.572. The specificity and sensitivity were 0.794 and 0.522, respectively (Figure 2). The C-index of the external validation cohort was 0.700 (95%CI:0.631–0.768), and the optimal cut-off value was 0.528. The specificity and sensitivity were 0.667 and 0.669, respectively (Figure 3), and the C-index of the internal validation cohort was 0.639 (0.638–0.643).

**Figure 4:**
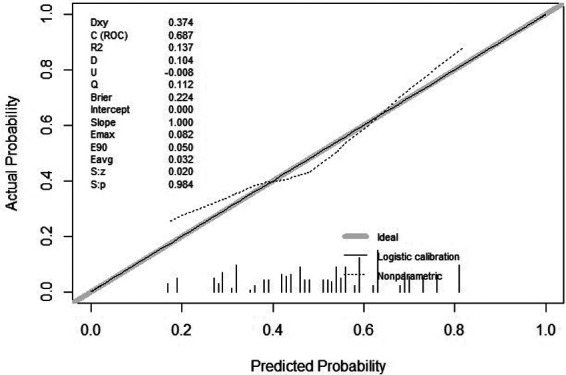
Calibration curve for development cohort of tibial plateau fracture combined with meniscus injury.

**Figure 5:**
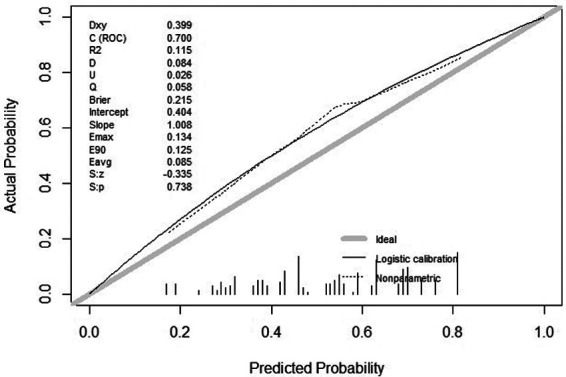
Calibration curve for external validation cohort of tibial plateau fracture combined with meniscus injury.

**Figure 6:**
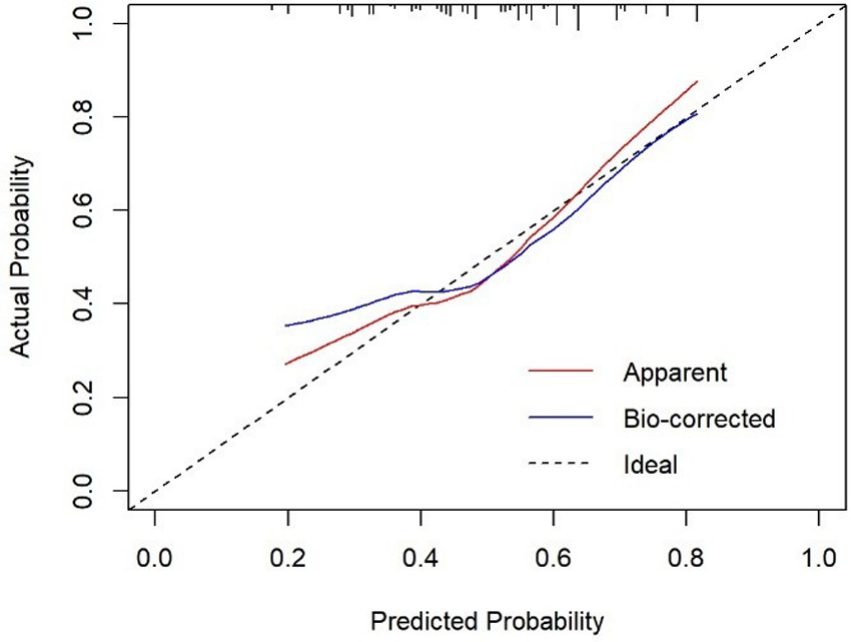
Calibration curve for internal validation cohort of tibial plateau fracture combined with meniscus injury.

In the calibration figures for the modeling, internal validation, and external validation groups, the prediction curve fitted the reference line well, indicating that the risk predicted by the model was consistent with the actual risk of meniscal injury. The H-L test results of the development and validation cohorts were *P *= 0.120 (*χ*^2^*^ ^*= 14.074) and *P *= 0.216 (*χ*^2^*^ ^*= 11.961), respectively, indicating that the model had good predictive ability and a high level of calibration (Figures 4–6).

**Figure 7:**
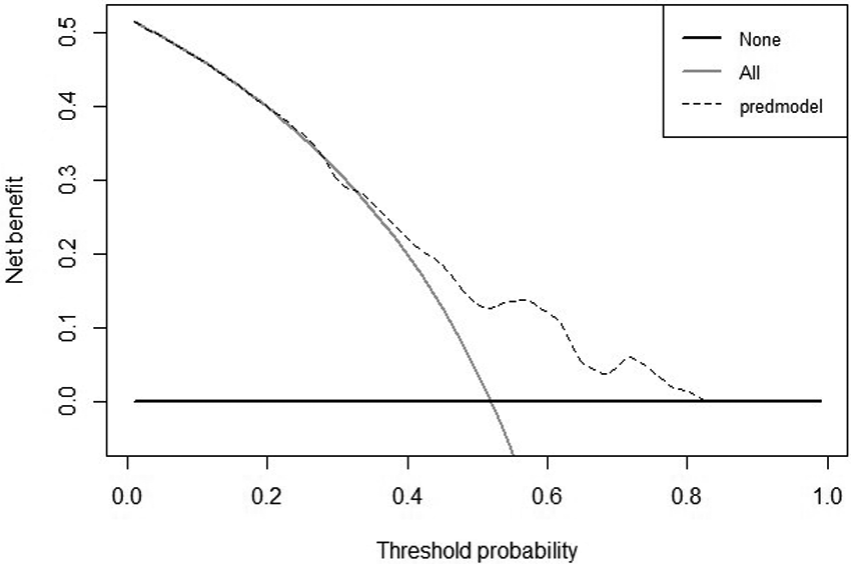
DCA curve for development cohort of tibial plateau fracture combined with meniscus injury.

**Figure 8:**
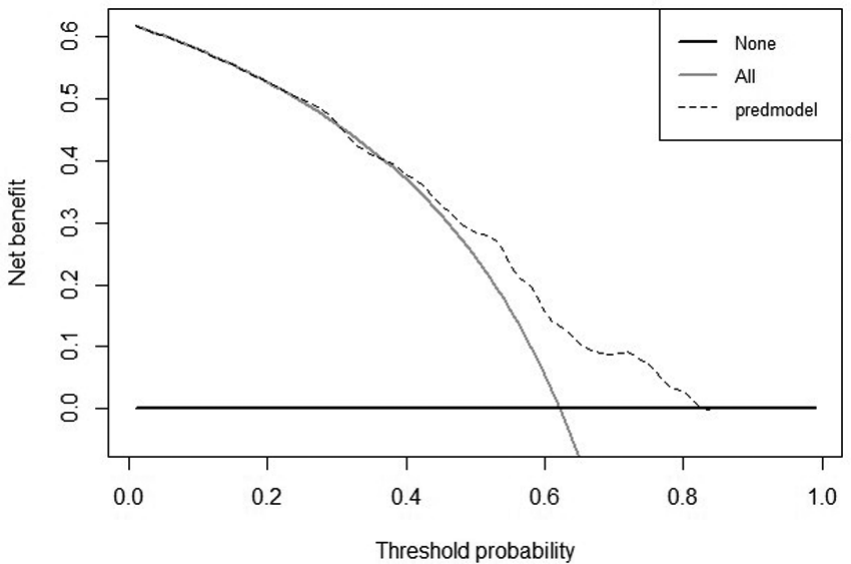
DCA curve for validation cohort of tibial plateau fracture combined with meniscus injury.

According to the DCA curve in the development group, the clinical effectiveness is best and the treatment has a higher net benefit, when the threshold probability is in the range of 0.40 to 0.82 (Figures 7, 8).

### Presentation of clinical prediction model

**Figure 9:**
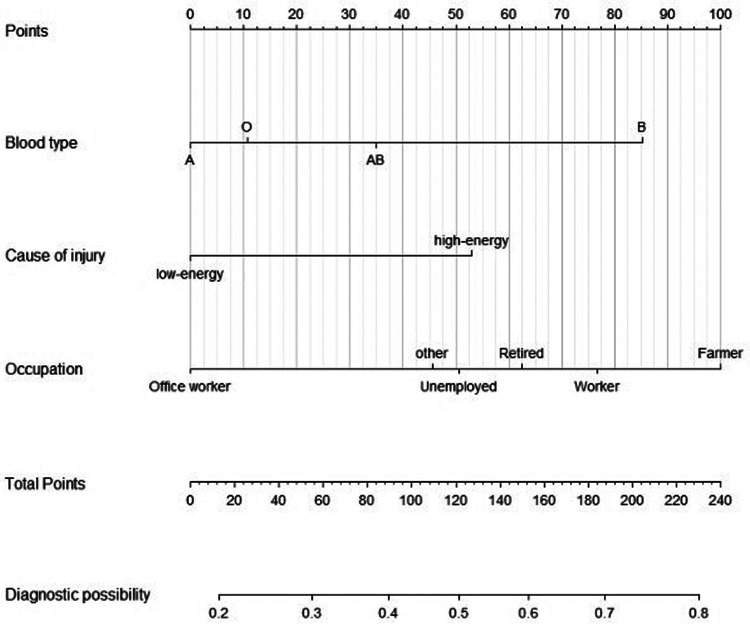
Nomogram for tibial plateau fracture combined with meniscus injury.

The “lrm” function in the Rms package of R language was used to establish the Logistic regression model with the above three factors. The “Plot” function was used to further draw the nomogram, and the results of the clinical prediction model were visualized (Figure 9). A nomogram can be used to determine the risk of tibial plateau fracture combined with meniscal injury, according to the relevant variables of individuals in practice.

For example, a worker with blood type B suffered a tibial plateau fracture in the spring when he fell from a scaffolding that was 3 m high. The corresponding score was obtained on the nomogram based on the values of each factor. When the scores for each factor were summed, the risk of meniscal injury in this patient was 0.765.

## Discussion

Tibial plateau fracture combined with meniscal injury can cause serious damage to knee joint function. At present, meniscal injury is usually judged by knee MRI, which has a certain false-positive rate (14). Therefore, it is critical to take preventive measures against risk factors and high-risk groups that may develop meniscal injury. In this study, we developed and validated a clinical model to predict the risk of meniscal injury in patients with tibial plateau fractures. Through univariate and multivariate analyses, we included the selected variables in the model, and the model evaluation showed good discrimination, calibration, and clinical validity. According to the nomogram, occupation was the most important predictor, followed by blood type and injury mechanism.

In the current study, when compared with farmers, being office workers was a protective factor for tibial plateau fracture combined with meniscus injury. This may be related to the lower daily exercise and lower activity levels of office workers. This finding is similar to those of previous studies that showed that excess exercise is a known risk factor for meniscal injury (15, 16). Repeated bending and crouching can cause frequent compression and friction of the meniscus, resulting in minor trauma. When the long-term injury accumulation exceeds the physiological capacity, the meniscus will undergo pathological changes in tissue composition and structure, and even tears, which will affect function (17, 18). Office workers work does not involve heavy physical strength and endurance activities, as such there is less joint wear, and a lower incidence of meniscus injury. Conversely, farmers work involves heavy physical labor, endurance activities, and often the need to carry out activities which strain the knee joint such as squatting or weightlifting. Such activities can easily cause acute or chronic meniscus injury. Some studies have found that compared with the general population, the activity levels of farmers, professional athletes, and soldiers are significantly higher, and the incidence of meniscus injury is also markedly increased (19, 20). In addition to the lower level of activity, the higher level of education and income of the office workers may be another factor. Education level is closely related to health literacy. Typically, the higher the education level, the better the economic condition, higher the health literacy, and healthier the lifestyle and behavior (21, 22). The average education level and per capita disposable income of office workers was higher than those of farmers. Moreover, office workers are more likely to perform reasonable exercises in their daily life and pay attention to the protection of the knee joints, thus lowering the incidence of meniscal injury. These findings are similar to those of previous studies. Lee (23) found that the incidence of knee osteoarthritis was higher in people with low income, low education level, non-management jobs or unemployed. Moreover, the study showed the correlation between low education level and knee osteoarthritis was the strongest.

The findings from this study showed that tibial plateau fracture patients with blood type B were more likely to have meniscal injury complications than those with blood type A. The relationship between blood type and the prognosis of cardiovascular disease and cancer has been well established (24–28). However, a direct link between blood type and meniscal injury has not been proven. These findings are similar to those of previous studies that showed that ABO blood type is associated with personality characteristics. People with blood type B tend to be enthusiastic, active and explorative (29, 30). Daily activities are frequent, and they prefer short-range, medium-high-intensity exercise, which results in repeated flexion and extension of the knee joint over a prolonged time, frequent stimulation, and excessive load on the meniscus. Therefore, blood type may affect individual behavior by influencing personality which subsequently affects tibial plateau fracture combined with meniscal injury, this is consistent with the results of this study.

Another issue elucidated in this study was that patients with tibial plateau fractures with high-energy injuries are more likely to have meniscal injuries than those with low-energy injuries. High energy injury refers to the injury caused by falling from a height >1 m, the strong impact and extrusion of electric vehicles or motor vehicles, etc (31). When the human body is impacted, the cushioning effect of the meniscus can reduce the impact force on the articular surface and subsequent body injury. When falling from a height, in order to share the pressure on the femur, the meniscus bares a huge load, resulting in serious deformation of the meniscus and easily causing a crush injury. When there is a traffic accident, the knee joint often experiences excessive flexion or joint torsion, resulting in meniscal tear injury (4). Chen (32) reported that traffic accidents were the main cause of posterior cruciate ligament injuries, particularly motorcycle accidents. Ligament injuries are also associated with meniscal tears. However, Shamrock (33) found that the mechanism of high-energy injury was associated with increased cartilage injury rates, but not with meniscal injury rate.

Other factors for tibial plateau fracture combined with meniscal injury were also identified in the different subgroups during the current study. Many studies have shown that a high BMI is a known risk factor for degenerative meniscal injury, and most of the people in this study had high-energy injuries. Therefore, it was concluded that BMI is not a risk factor for tibial plateau combined with meniscal injury. Furthermore, in this study it was found that tibial plateau fracture classification is not related to meniscal injury, a finding that is consistent with other investigations showing that there was no difference in the distribution of meniscal injuries among different types of tibial plateau fractures (34–36). Moreover, results showed age was not a risk factor for tibial plateau combined with meniscal injury, and with increasing age, the meniscus undergoes degenerative changes, which can be damaged by a slight impact (37–39). On the other hand, young people engage in more vigorous sports which include bouncing, twisting, and collision motions, thus increasing the risk of meniscus injury (40, 41). This study concluded that sex and ligament injury were not risk factors for tibial plateau combined with meniscal injury, which is consistent with the results of many existing studies (42–44).

There are some limitations in this study. Firstly, it is a retrospective study with certain information bias. Secondly, fracture data from one hospital is unrepresentative, Thirdly, the data of fracture patients in 2022 in the development cohort only included patient data from January to June, resulting in a difference between the time range and the validation cohort. The relationship between time factors and complications of meniscal injury may be biased. As such, further large-sample, multicenter prospective studies are needed to increase the time and space range of data acquisition to obtain a more comprehensive and accurate data basis.

## Conclusions

Farmer occupation, blood type B and high-energy injuries are independent risk factors for tibial plateau fracture complicated with meniscal injury. Based on this, a clinical prediction model was established and evaluated. This model can be used to predict the menisci of patients with tibial plateau fractures. This can provide guidance for orthopaedic surgeons to make targeted preoperative examinations and surgical plans.

## Data Availability

The original contributions presented in the study are included in the article, further inquiries can be directed to the corresponding author/s.
