# Corrigendum: Prediction model for tibial plateau fracture combined with meniscus injury

**DOI:** 10.3389/fsurg.2024.1433731

**Published:** 2024-06-07

**Authors:** Hongzhi Lv, Wenjing Li, Yan Wang, Wei Chen, Xiaoli Yan, Peizhi Yuwen, Zhiyong Hou, Juan Wang, Yingze Zhang

**Affiliations:** Department of Orthopedic Surgery, The Third Hospital of Hebei Medical University, Shijiazhuang, China

**Keywords:** tibial plateau fracture, meniscus injury, risk factors, prediction model, nomogram

**A Corrigendum on**
Prediction model for tibial plateau fracture combined with meniscus injury Lv H, Li W, Wang Y, Chen W, Yan X, Yuwen P, Hou Z, Wang J and Zhang Y. (2023) Prediction model for tibial plateau fracture combined with meniscus injury. Front Surg. 10:1095961. doi: 10.3389/fsurg.2023.1095961

## Incorrect funding

In the published article, there was an error in the Funding statement. The name and the number of one fund were incorrectly written as follows “Natural Science Foundation of Hebei Province (CN)—for Distinguished Young Scholars (H2021206329)”.

The correct Funding statement appears below.

